# Association between Vitamin D and Circulating Lipids in Early Childhood

**DOI:** 10.1371/journal.pone.0131938

**Published:** 2015-07-15

**Authors:** Catherine S. Birken, Gerald Lebovic, Laura N. Anderson, Brian W. McCrindle, Muhammad Mamdani, Sharmilaa Kandasamy, Marina Khovratovich, Patricia C. Parkin, Jonathon L. Maguire, TARGet Kids! collaboration

**Affiliations:** 1 Pediatric Outcomes Research Team (PORT), Division of Paediatric Medicine, Department of Paediatrics, The Hospital for Sick Children, Toronto, Ontario, Canada; 2 Department of Pediatrics, Faculty of Medicine, University of Toronto, Toronto, Ontario, Canada; 3 Institute for Health Policy, Management and Evaluation, University of Toronto, Toronto, Ontario, Canada; 4 The Applied Health Research Centre of the Li Ka Shing Knowledge Institute of St. Michael’s Hospital, Toronto, Ontario, Canada; 5 Cardiology Division, The Hospital for Sick Children, Toronto, Ontario, Canada; 6 Leslie Dan Faculty of Pharmacy, University of Toronto, Toronto, Ontario, Canada; 7 Department of Pediatrics, St. Michael’s Hospital, Toronto, Ontario, Canada; Hunter College, UNITED STATES

## Abstract

Vitamin D is associated with established cardiovascular risk factors such as low density lipoprotein (LDL) in adults. It is unknown whether these associations are present in early childhood. To determine whether serum 25-hydroxyvitamin D (25(OH)D) is associated with serum non-high density lipoprotein (non-HDL) cholesterol during early childhood we conducted a cross-sectional study of children aged 1 to 5 years. Healthy children were recruited through the TARGet Kids! practice based research network from 2008-2011 (n=1,961). The associations between 25(OH)D and non-fasting non-HDL cholesterol (the primary endpoint), total cholesterol, triglycerides, HDL, and low density lipoprotein (LDL) cholesterol, were evaluated using multiple linear regression adjusted for age, sex, skin pigmentation, milk intake, vitamin D supplementation, season, body mass index, outdoor play, and screen time. Each 10 nmol/L increase in 25(OH)D was associated with a decrease in non-HDL cholesterol concentration of -0.89 mg/dl (95% CI: -1.16,-0.50), total cholesterol of -1.08 mg/dl (95%CI: -1.49,-0.70), and triglycerides of -2.34 mg/dl (95%CI: -3.23,-1.45). The associations between 25(OH)D and LDL and HDL were not statistically significant. 25(OH)D concentrations were inversely associated with circulating lipids in early childhood, suggesting that vitamin D exposure in early life may be an early modifiable risk factor for cardiovascular disease.

## Introduction

Cardiovascular disease is the leading cause of mortality in the United States and Canada and places the greatest burden of any disease on health care systems.[1] Multiple lines of evidence support atherosclerosis beginning at a young age [2] and that cardiovascular risk factors such as abnormal serum lipids present during childhood track well into adulthood.[3] Although the Institute of Medicine (IOM) concluded in 2011 that there was insufficient evidence to support a role of vitamin D beyond bone health,[4] it has been widely hypothesized that vitamin D may influence the development of cardiovascular disease. The vitamin D receptor is found in all cardiovascular cell types, including blood vessels, and the active form of vitamin D, 1, 25-dihydroxyvitamin D, improves endothelial cell function through decreased inflammation, and modulates proliferation and differentiation of cardiomyocytes.[5,6]

In adults, observational studies have found lower 25-hydroxyvitamin D (25(OH)D) concentrations are associated with metabolic syndrome, obesity, hypertension, diabetes, myocardial infarction, stroke and overall cardiovascular death.[7,8] Results of a Mendelian randomization study in adults suggested that higher vitamin D may be associated with more favorable lipid profiles.[9] Recent findings from randomized controlled trials of vitamin D and serum lipids in adults have been inconsistent, with some studies not demonstrating a beneficial effect of vitamin D.[10,11] In older children and adolescents, lower 25(OH)D concentrations have been associated with traditional cardiovascular disease risk factors including obesity, elevated systolic blood pressure, decreased high density lipoprotein (HDL) cholesterol, and insulin resistance.[12,13] However, there are few studies of vitamin D and serum lipids specifically focused on young children. If vitamin D is associated with serum lipids in early childhood, this may provide an opportunity for early life interventions to reduce cardiovascular risk.

One important marker for cardiovascular disease risk in childhood is serum non-HDL cholesterol concentration (or total cholesterol minus HDL).[14] Higher non-HDL cholesterol during childhood (ages 3–18 years) has been associated with an adult measure of atherosclerosis (carotid artery intima-media thickness)[15] and atherosclerosis among 15–34 year olds who died traumatically.[16] Further, non-HDL cholesterol has been identified as a better childhood predictor of adult dyslipidemia and non-lipid cardiovascular risk factors than LDL cholesterol.[2] Non-HDL cholesterol has a major advantage over other serum lipids as it is not dependent on fasting status, making it feasible to measure in young children in whom fasting is not practical.[17] Serum non-HDL cholesterol is the dyslipidemia universal screening test currently recommended for children by the National Heart, Lung and Blood Institute and endorsed by the American Academy of Pediatrics.[14]

Given the association between vitamin D and adult cardiovascular disease, we hypothesized that there may be an association between vitamin D and an early life marker of cardiovascular risk, non-HDL cholesterol. The primary objective of this study was to determine whether serum 25(OH)D is associated with serum non-HDL cholesterol concentration during early childhood. The secondary objectives were to evaluate whether 25(OH)D is associated with other traditional serum markers of cardiovascular risk including non-fasting total cholesterol, triglycerides, HDL and low density lipoprotein (LDL) during early childhood.

## Materials and Methods

### Subjects and design

A cross sectional study was conducted of children between 1 and 5 years of age attending scheduled well-child visits through The Applied Research Group for Kids (TARGet Kids!) between December 2008 and June 2011. TARGet Kids! is a primary care practice based research network (www.targetkids.ca) in Toronto, Canada and has been described previously.[18] Children were excluded if they had any chronic illnesses (excluding asthma), severe developmental delay, were on a medication known to alter vitamin D metabolism (e.g., phenobarbitol) or had a gestational age <32 weeks.

### Ethics statement

This study was approved by the Research Ethics Board at the Hospital for Sick Children, Toronto, Ontario, and St. Michael’s Hospital, Toronto, Ontario. Written consent was obtained from parents of all participating children.

### Subject recruitment and data collection

Study participants were recruited by research personnel who were embedded in seven participating pediatric and family medicine practices. Survey data were collected through a standardized parent-completed nutrition and health questionnaire [18]. Anthropometric measurements including height and weight were collected by trained research assistants using standardized instruments and non-fasting venous blood sampling was collected at the primary care practices. Specimens were sent daily to Mount Sinai Services Laboratory in Toronto, Ontario (www.mountsinaiservices.com). Medidata RAVE (Medidata Solutions Inc. http://www.mdsol.com/) was used as the secure electronic data capture system and data repository for all TARGet Kids! data.

### Exposure and outcome variables

Vitamin D was measured as total 25(OH)D from serum samples using a competitive two-step chemiluminescence assay (Diasorin LIAISON 25(OH)D TOTAL). This method has demonstrated an intraassay imprecision of 7.2% at a concentration of 213 nmol/L and an interassay imprecision of 4.9% at 32 nmol/L, 8.9% at 77 nmol/L and 17.4% at 213 nmol/L, values which are well within acceptable limits[19,20].

The primary outcome for this study was serum non-HDL concentration which was calculated as total cholesterol minus HDL. Secondary outcomes were non-fasting total cholesterol, triglycerides, HDL and LDL. There is some evidence that fasting may not be necessary for cholesterol screening in children.[21] Non-fasting serum lipids were measured using a flurometric assay on the Roche Modular P Chemistry Analyzer calibrated to current Centers for Disease Control and Prevention (CDC) guidelines. Triglyceride data was positively skewed and a log transformation was performed. All laboratory analysis was performed by the Mount Sinai Services Laboratory using standard procedures (http://www.mountsinaiservices.com/).

### Other variables

All potential confounders, hypothesized *a priori* to be associated with both 25(OH)D and serum lipids, and covariates known or suspected to affect 25(OH)D or serum lipids were measured. These included age, sex, season of blood draw (October-April versus May–September), vitamin D supplementation, daily volume of cow’s milk intake, daily minutes of outdoor play, daily minutes of screen time, body mass index (BMI), and skin pigmentation. Child’s vitamin D supplementation and typical daily cow’s milk intake (fortified with 100 IU vitamin D per 250 ml cup in Canada) were determined from the questionnaires. Weight and standing height (or length for children under 2 years old) were measured by trained research assistants. BMI was calculated as weight in kilograms divided by the height in meters squared. BMI z-scores were calculated using World Health Organization (WHO) growth standards[22] as recommended for this age group.[23] Skin pigmentation was recorded by a research assistant using the Fitzpatrick scale.[24]

### Statistical analysis

Descriptive statistics were calculated for the main outcome, exposure, and covariates for children with and without blood measures. Multiple linear regression was used to evaluate the associations between 25(OH)D and our primary outcome, serum non-HDL concentration, and each of our secondary outcomes, total cholesterol, triglycerides, HDL and LDL. All outcomes were normally distributed with the exception of triglycerides which was log transformed in the regression analysis. All models were checked using residual plots and assumptions were valid. All adjusted models included all of the pre-specified, clinically relevant covariates described above. Multi-collinearity was examined using correlation matrices and variance inflation factors. Since triglycerides were log transformed, the effect sizes for triglycerides were reported at the mean value by retransformation of the parameter estimates in conjunction with a Duan’s smearing estimate.[25]

Potential interactions between 25(OH)D and outdoor play time, vitamin D supplementation, zBMI, age, skin pigmentation and milk intake were explored, using a joint test for interaction; if the joint *P* > 0.3 no further testing was considered.[26] A false discovery rate controlling procedure was used to adjust the statistically significant p-value for testing of secondary outcomes.[27] Multiple imputation was implemented using predictive mean matching with fifty datasets used for each model.[26] Data were analyzed using the R project for statistical computing, version 2.14.1 (http://www.R-project.org/) and statistical significance was determined by a 2-sided *P* < 0.05, after adjustment for multiple testing for the secondary outcomes.

## Results

Of the 3524 children who consented to participate, non-fasting venous blood sampling was obtained in 1961 (56%) children who were included in the analysis. Children with blood sampling were slightly older, more likely to be recruited during the winter and more likely to be receiving a vitamin D supplement. Among children with blood and included in the analysis, mean daily cow’s milk intake was 452 mL and 56% of children were regularly consuming a vitamin D supplement (Table 1). Mean 25(OH)D was 85 nmol/L (SD = 30) and mean non-HDL concentration was 110 mg/dL (SD = 26) (Table 1).

**Table 1:** Characteristics of Participants With and Without Blood in TARGet Kids!, 2008–2011.

Characteristic	Children with blood (Study Population) N = 1961	Children without blood N = 1570
	Mean (SD)	Mean (SD)
Age (months)	36 (18)	33 (17)
25(OH)D (nmol/L)	85 (30)	
Non-HDL (mg/dL)^a^	110 (26)	
Total Cholesterol (mg/dL)	158 (26)	
LDL (mg/dL)	87 (25)	
HDL (mg/dL)	48 (12)	
Triglycerides (mg/dL)	117 (62)	
Daily milk intake (mL)	452 (305)	431 (285)
Daily outdoor play time (min)	62 (56)	64 (64)
Daily Screen time (min)	79 (77)	78 (80)
BMI z score	0.21 (1.0)	0.20 (1.1)
	**N (%)**	**N (%)**
Sex, male	996 (51%)	817 (52%)
Daily vitamin D supplementation	1047 (56%)	619 (41%)
Season (Oct-April), n (%)	1020 (52%)	961 (61%)
BMI z-score		
Overweight (1.0–2.0)	372 (20%)	297 (21%)
Obese (>2.0)	86 (5%)	64 (5%)
Skin Pigmentation (Fitzpatrick)		
I–III (Lighter pigmentation)	1558 (85%)	1191 (86%)
IV–VI (Darker pigmentation)	276 (15%)	197 (14%)
Ethnicity		
European	1312 (71%)	1133 (73%)
East Asian	127 (7%)	109 (7%)
South/Southeast Asian	170 (9%)	127 (8%)
Other	253 (14%)	185 (12%)
Abnormal cut points^b^		
Non-HDL ≥145 mg/dL	169 (9%)	
Total Cholesterol ≥200 mg/dL	113 (6%)	
LDL ≥130 mg/dL	89 (5%)	
HDL <40 mg/dL	498 (26%)	
Triglycerides^c^ ≥100 mg/dL	998 (52%)	

^a^To convert from mg/dL to SI units (mmol/L) divide the results for non-HDL, Total Cholesterol, LDL and HDL by 38.6, and divide by 88.6 for triglycerides.

^b^Abnormal cut-point values for plasma lipid levels are from the National Cholesterol Education Program (NCEP) Expert Panel on Cholesterol Levels in Children. Non-HDL cholesterol values from the Bogalusa Heart Study are equivalent to the NCEP Pediatric Panel cut points for LDL cholesterol. [14]

^c^Triglyceride cut-point is from fasting sample recommendations.

In the fully adjusted model, a statistically significant association was observed between increased 25(OH)D and decreased non-HDL cholesterol (Table 2). Each 10 nmol/L increase in 25(OH)D was associated with a statistically significant decrease in non-HDL cholesterol of -0.89 mg/dl (95% CI: -1.16, -0.50.). Statistically significant covariates included age, sex, cow’s milk intake, and BMI z-score (Table 2). A simultaneous test of all hypothesized interactions was not statistically significant (*P* = 0.98).

**Table 2:** Adjusted Association Between 25-Hydroxyvitamin D (per 10 nmol/L increase) and Non-HDL (mg/dL) Among Children 1 to 5 Years of Age in TARGet Kids!, 2008–2011.

Variable	Adjusted Estimate (mg/dL)^a^	95% CI Lower Upper		p-value
25-hydroxyvitamin D (per 10 nmol/L)	-0.89	-1.16	-0.50	<0.0001
Age (per year)	-3.09	-3.86	-2.32	<0.0001
Sex (male)	-6.19	-8.51	-3.87	<0.0001
Daily vitamin D supplementation (yes vs. no)	2.32	-0.12	4.64	0.06
Cow’s milk (per cup/day)	1.55	0.39	2.32	0.002
Season (May-Sept vs. Oct-April)	0.00	-2.32	2.32	0.92
Daily outdoor play time (per 1 hour/day)	-0.77	-1.93	0.77	0.33
Screen time (per 1 hour/day)	0.77	-0.39	1.55	0.15
BMI Z-score (per unit)	1.16	0.12	2.32	0.03
Skin pigmentation (I-III vs. IV-VI)	3.10	-0.11	6.57	0.06

^a^To convert from mg/dL to SI units divide the results for non-HDL, Total Cholesterol, LDL and HDL by 38.6, and divide by 88.6 for triglycerides.

For our secondary analysis, each 10 nmol/L increase in 25(OH)D was associated with a decrease in non-fasting total cholesterol of 1.08 mg/dl (95% CI: 0.70, 1.49 mg/dl) and a decrease in non-fasting triglycerides of 2.34 mg/dl (95% CI: 1.45, 3.23 mg/dl) in the adjusted analysis (Table 3). After correcting for multiple hypothesis testing, statistically significant relationships between 25(OH)D and both LDL and HDL were not identified. The results of the fully adjusted analysis were not substantially different than the unadjusted analysis (Table 3).

**Table 3:** Unadjusted and Adjusted Association Between 25-Hydroxyvitamin D (per 10 nmol/L increase) and Non-Fasting Serum Lipids (mg/dL) Among Children 1 to 5 Years of Age in TARGet Kids!, 2008–2011.

	Unadjusted			Adjusted^a^		
	Estimate (mg/dL)	95% CI	p-value^b^	Estimate (mg/dL)	95% CI	p-value^b^
Non-HDL	- 0.70	-1.1, -0.50	0.0004	- 0.89	-1.16,-0.50	< 0.0001
Total Cholesterol	- 0.89	-1.3, -0.50	< 0.0001	- 1.08	-1.49, -0.70	< 0.0001
LDL	-0.19	-0.58, 0.15	1.00	-0.35	-0.70, 0.02	0.256
HDL	- 0.19	-0.38, -0.04	0.048	- 0.19	-0.36, -0.02	0.078
Triglycerides^c^	- 2.66	-3.54, -1.68	< 0.0001	- 2.34	-1.45, -3.23	< 0.0001

^a^Adjusted for age, sex, season, vitamin D supplementation, daily volume of cow’s milk intake, daily minutes of outdoor play, daily minutes of screen time, zBMI, and skin pigmentation.

^b^P-values for secondary objectives adjusted for multiple testing using a false discovery rate controlling procedure correction. Statistical significance is defined as an adjusted P-value <0.05.

^c^Triglyceride values were log transformed for analysis and back transformed results are presented.

## Discussion

Our study is the largest study to date examining the association between 25(OH)D and lipids in young children. Our findings suggest that vitamin D, as measured from serum 25(OH)D concentrations, is associated with non-HDL cholesterol, as well as non-fasting triglycerides and total cholesterol during early childhood, surrogate markers for adult cardiovascular disease. These associations were present even after taking into account numerous known or suspected confounders including BMI, cow’s milk intake and physical activity.

To our knowledge, only one previous study has evaluated the association between vitamin D and non-HDL in a small sample (n = 171) of children with a broad age range from 2–18 years (few young children), and no association was observed.[28] Our findings of inverse associations between 25(OH)D and both non-fasting triglycerides and total cholesterol are consistent with one other study among 255 infants aged 9 months[29]; however, similar to our study, samples were not collected after overnight fast and thus the association may reflect a 25(OH)D gradient in the short-term metabolism of lipids. Other studies of 25(OH)D and triglycerides in older children or adolescents have largely been null [12,13,30,31,32,33] and studies of 25(OH)D and total cholesterol have been inconsistent.[13,29,30,31,34]

Our finding of no association between 25(OH)D and HDL among young children is consistent some of the previous literature [30,35]; however, other studies, largely in adolescent populations, have identified a BMI independent positive association between 25(OH)D and HDL, consistent with the hypothesis that vitamin D is associated with a more favorable lipid profile.[12,13,28,31,32,33,34,36] Only one study, among infants 9 months of age, identified an inverse trend between 25(OH)D and HDL.[29] Most of the studies of 25(OH)D and LDL in older children or adolescents have been non-significant which is consistent with our observation.[12,31,32,34,35]

The clinical significance of elevated serum non-HDL cholesterol in early childhood is currently under study but existing evidence suggests that non-HDL cholesterol levels are associated with later cardiovascular disease.[15,16] The National Heart and Blood Institute currently recommends non–HDL as the key measure for universal screening children for cardiovascular risk as it appears to be a good predictor of adult lipid and non-lipid cardiovascular risk factors and is not dependent on fasting status, an important practical consideration for young children where fasting is challenging[14]; there is, however, evidence that fasting may not be necessary for lipid screening in children and healthy adults.[21,37]

A strength of our study is the relatively large sample of young healthy children with blood measures recruited from a population based setting. Few studies have collected blood on healthy children less than 5 years of age recruited from primary care. Further, detailed information from standardized questionnaires and physical measures allowed for the adjustment of multiple biologically plausible confounders. Some may argue that the absolute effect of 25(OH)D on non-HDL cholesterol appears small, however at a population level this relationship may have important implications on the cumulative risk of cardiovascular disease over a the life course.

Limitations of this study include the cross-sectional study design and causation cannot be inferred from the identified associations. Future longitudinal studies of 25(OH)D and non-HDL cholesterol are needed. Residual confounding from unmeasured confounders is possible. Although cow’s milk consumption was measured, other measures of lipid intake such as the consumption of fast foods were not measured which may have influenced both 25(OH)D and serum lipids. We also cannot rule out the possibility of selection bias; it is possible that children with unfavorable lipid profiles and lower 25(OH)D are less likely to participate in the study. However, given that children were recruited from their primary care provider all children had an equal opportunity to participate. Child height and weight were collected by trained research assistants and we did adjust for zBMI, although detailed measures of adiposity (i.e., DXA scans) on these young children may be preferable. This study included children from a single geographic location situated at 43°N which is similar latitude to several other large North American cities, but may not be generalizable to other populations.

## Conclusions

We have identified an association between higher 25(OH)D and lower non-HDL cholesterol. With a potentially long duration of exposure to cardiometabolic risk factors, identifying modifiable factors such as 25(OH)D that might influence cardiovascular risk factors during early childhood could have long-term health benefits. Randomized controlled trials are needed to determine if modifying 25(OH)D during early childhood has a causal effect on non-HDL cholesterol concentration and other serum lipids. If the association between 25(OH)D and lipids is causal, this may identify early life interventions for cardiovascular disease prevention. In the context of lifetime risk of cardiovascular disease associated with increased cholesterol beginning in early childhood, these findings may have important public health implications should they prove causal.
